# Adapting and testing of DeprEnd EMDR therapy for major depressive disorder: a study protocol of mixed method randomized controlled trial

**DOI:** 10.1186/s13063-026-09543-4

**Published:** 2026-02-16

**Authors:** Anwar Khan, Amalia bt Madihie, Maqsood Haider, Sajjad Haider, Rawaiz Khan, Ali Bahadar

**Affiliations:** 1 Faculty of Cognitive Sciences and Human Development, Universtiti Malaysia Sarawak, Kota Samarahan, Sarawak Malaysia; 2 Department of Management Sciences and Psychology, Khushal Khan Khattak University Karak, Karak, Pakistan; 3 Department of Management Sciences, FATA University, Kohat, Pakistan; 4 Chemical Engineering Department, College of Engineering, King Saud University, P.O. Box 800, Riyadh, 11421 Saudi Arabia; 5 Department of Restorative Dental Sciences, College of Dentistry, King Saud University, Riyadh, 11545 Saudi Arabia; 6 Department of Chemical and Materials Engineering, King Abdulaziz University, Rabigh, 21911 Saudi Arabia; 7 King Salman Center for Disability Research, Riyadh, 11614 Saudi Arabia

**Keywords:** Eye movement desensitization and reprocessing, Major depressive disorder, Cultural and methodological adaptations, Online psychotherapy, Pakistan

## Abstract

**Background:**

Major depressive disorder (MDD) is a significant contributor to the global burden of disease, with a high prevalence in Pakistan. MDD is treatable, and eye movement desensitization and reprocessing (EMDR) therapy is recognized as an effective treatment worldwide. However, EMDR therapy, rooted in Western psychological frameworks, may require cultural and methodological adaptation to fit clients’ needs in Pakistan. Notably, there is a lack of research on adapting EMDR therapy in Pakistan, and scarce studies on its effectiveness through online modalities. To bridge these research gaps, this study has two aims: first to develop an adapted DeprEnd EMDR therapy protocol in Pakistan; second, to test its feasibility, relevance, and clinical effectiveness across in-person and online modalities.

**Methods:**

This research adopts a mixed-mode exploratory sequential randomized controlled trial design. In the first phase, an exploratory qualitative approach will be utilized to culturally and methodologically adapt DeprEnd EMDR therapy protocol through narrative review and focus group discussion. Data will be qualitatively analyzed. This phase has been previously reported in a separate peer-reviewed publication. In the second phase, a randomized controlled trial design will be used to test the feasibility, relevance, and clinical effectiveness of the adapted DeprEnd EMDR therapy protocol. Initially, it will be pilot tested among 25 handful clients, and later an estimated sample of 80 clients will be selected to test it on a large scale. Clients will be randomized via a covariate-adaptive technique to in-person and online arms with a 1:1 ratio. Symptom-related data will be collected at the baseline, midpoint, post-treatment, and follow-up stages. Data will be analyzed using a combination of univariate and multivariate statistics.

**Discussion:**

This research conducts the first known scientific adaptation and clinical testing of the DeprEnd EMDR therapy protocol in Pakistan. Through systematic adaptation and assessment of its feasibility and clinical effectiveness, this protocol is positioned to be scalable, showing potential for broader dissemination across South Asia. It contributes a vital framework for culturally sensitive mental health interventions that bridge global evidence-based practices and local sociocultural needs. This study paves the way for collaborative efforts to optimize trauma-focused psychotherapies in low-resource contexts, thus promoting health equity in the region.

**Trial registration:**

The initial study protocol was registered in the ClinicalTrials.gov database under registration no: NCT-06439043. Last Updated:01/21/2025 and Initial Release:05/27/2024.

**Supplementary Information:**

The online version contains supplementary material available at 10.1186/s13063-026-09543-4.

## Administrative information

Note: the numbers in curly brackets in this protocol refer to SPIRIT checklist item numbers. The order of the items has been modified to group similar items (see http://www.equator-network.org/reporting-guidelines/spirit-2013-statement-defining-standard-protocol-items-for-clinical-trials/).

**Table:** 

Title {1}	Adapting and Testing of DeprEnd EMDR Therapy for Major Depressive Disorder: A Study Protocol of Mixed Method Randomized Controlled Trial
Trial registration {2a and 2b}	ClinicalTrials.gov database under Registration No: NCT-06439043.
Protocol version {3}	Version 2.0 finalized on January 15, 2025.
Funding {4}	The EMDR Research Foundation, USA, initially funded this study through Dr. Sandra Wilson’s Dissertation Research Grant. Subsequently, the King Salman Center for Disability Research, Saudi Arabia (funder ID: 10.13039/501100019345; Research Group No. KSRG-2024-116) provided support for marketing and publication expenses related to this research.
Author details {5a}	^1^Postgraduate Scholar, Faculty of Cognitive Sciences and Human Development, Universtiti Malaysia Sarawak, Kota Samarahan, Sarawak, Malaysia. ^2^Assistant Professor, Department of Management Sciences and Psychology, Khushal Khan Khattak University Karak, Pakistan. ^3^Associate Professor, Faculty of Cognitive Sciences and Human Development, Universtiti Malaysia Sarawak, Kota Samarahan, Sarawak, Malaysia. ^4^ Department of Management Sciences, FATA University. ^5^ Chemical Engineering Department, College of Engineering, King Saud University, P.O. Box 800, Riyadh, 11421, Saudi Arabia. ^6^Department of Restorative Dental Sciences, College of Dentistry, King Saud University, Riyadh, 11545, Saudi Arabia. ^7^Department of Chemical and Materials Engineering, King Abdulaziz University, Rabigh, 21911, Saudi Arabia. ^8^King Salman Center for Disability Research, Riyadh 11614, Saudi Arabia.
Name and contact information for the trial sponsor {5b}	Anwar Khan, Assistant Professor, Department of Management Sciences and Psychology, Khushal Khan Khattak University Karak, Pakistan.
Role of sponsor {5c}	The study sponsor also serves as the principal investigator of this research. However, the funders had no involvement in the study design; data collection, management, analysis, or interpretation; report writing, or the decision to submit the findings for publication.

## Introduction

### Background and rationale {6a}

#### The state of mental health in Pakistan

In Pakistan, mental health is shaped by socioeconomic, cultural, infrastructural, and political conditions, leading to high rates of untreated mental health conditions—such as depression and anxiety—particularly among marginalized groups [1]. Recent statistics show that around 24 million people in Pakistan need mental health care [2]. Moreover, literature reports a high prevalence of depressive disorders in Pakistan. A recent study published in the Lancet reported a 79% prevalence of depression in Pakistan [3]. Similarly, a meta-analysis of 7652 university students across 26 studies published in Pakistan identified a pooled depressive symptom prevalence of 42.70% (95% CI: 34.8–50.9%) [4]. Furthermore, studies by Khan et al. [4], Khattak et al. [5], Ullah et al. [6] and Sana [7] have reported a significant prevalence of depressive symptoms in Pakistan. However, limited professional health resources in the country impact treatment accessibility and quality of care for individuals seeking mental health services. This issue is exacerbated by a lack of mental health awareness and stigma surrounding mental illness in the country [8]. These prevalence findings clarify the local epidemiology of depression in Pakistan and highlight a critical gap: the need for local research to culturally adapt evidence-based treatments. Addressing this gap is vital for improving domestic mental healthcare and contributing effective models to global psychiatry.

#### Treatment of major depressive disorder

Major depressive disorder (MDD) is treatable either through psychotherapy or pharmacotherapy. However, findings of recent systematic reviews revealed that psychotherapy is more efficacious, particularly in the long-term treatment of MDD [8–10]. Furthermore, research indicates that pharmacotherapy is associated with side effects [11] and withdrawal symptoms [12]. Given these risks, pharmacotherapy should be prescribed judiciously. Psychotherapy may serve as a beneficial first line or adjunctive treatment for depression.

Among the most recognized evidence-based psychotherapies are eye movement desensitization and reprocessing (EMDR) [13] and cognitive behavioral therapy [14]. CBT is a well-established approach with decades of research supporting its efficacy. EMDR therapy, though developed more recently, has gained substantial empirical support, particularly for trauma. EMDR therapy is an eight-phase psychotherapy that is based on Adaptive Information Processing theory that targets maladaptively stored memories, and via its bilateral stimulation technique, it reprocesses such memories to a more adaptive state [15]. However, as a newer modality, EMDR requires further investigation to clarify its full range of applications. In Pakistan, CBT was introduced in the early 1990s. Since then, CBT has been widely acknowledged for diverse mental health disorders across the country [16]. However, CBT still faces limitations including issues related to accessibility in rural clinical settings, cultural mismatches, and systemic issues like lack of research funding [17]. In contrast, EMDR therapy remains a relatively emerging intervention in Pakistan. It was first introduced in Pakistan via the EMDR Europe Humanitarian Assistance Programs Project after the 2005 earthquake [18]. EMDR therapy has undergone limited localized adaptation and lacks sufficient region-specific research to validate its efficacy and cultural appropriateness in Pakistan [19].

More specifically, the recently introduced DeprEnd EMDR therapy protocol for depression [17, 18] has primarily been tested in developed countries, such as Germany [20]. The DeprEnd EMDR differs from the standard EMDR therapy protocol since it systematically targets depressive core memory networks rather than focusing primarily on discrete traumatic events [21]. However, to our knowledge, no published studies have clinically tested the DeprEnd EMDR therapy protocol in Pakistan. This significant research gap highlights the urgent need for rigorous scientific research in the Pakistani context. Addressing this gap would not only advance local trauma care but also enrich the global EMDR evidence base through diversification.

#### The necessity of adapting and testing EMDR therapy in Pakistan

EMDR therapy was developed in the USA and is grounded in Western psychological frameworks [22]. Its clinical efficacy has primarily been established in Western countries such as the UK [23], the USA [24], and Australia and New Zealand [25], and other counties including Turkey [25], Japan [26], and China [27]. In Pakistan, relatively few studies have assessed the efficacy of EMDR therapy. Early work by Ali and Rana [28] documented clinically meaningful reductions in post-traumatic stress symptoms among survivors of the 2005 earthquake using EMDR. Subsequent case-based investigations by Bilal et al. [29] and Muhammad Sami et al. [30] reported symptom improvement in patients with complex post-traumatic stress presentations, suggesting the potential utility of EMDR beyond standard PTSD cases, albeit within small and methodologically limited samples. More recently, Khan, Madihie et al. [31] conducted a full-scale randomized controlled trial comparing EMDR with cognitive behavioral therapy for PTSD in Pakistan, demonstrating comparable or superior clinical outcomes for EMDR and providing the strongest empirical support to date for its effectiveness in the local context. Despite these contributions, the existing literature on EMDR therapy in Pakistan remains limited in scope, with most studies focusing on trauma-related disorders, relying on small samples or case-based designs, and lacking systematic cultural adaptation, implementation, and modality-comparison research. Moreover, past research predominantly focused on in-person delivery, leaving online modalities critically underexplored.

The existing literature is also significantly limited to cultural and methodological adaptations in EMDR therapy. Literature reports studies conducted in Cambodia [32] and Iran [33]; additionally, in Syria [34] and Germany [35], and finally, in five African countries [36]. While in Pakistan, studies are very scarce. Farrell et al. [37] recommended that Pakistani therapists must be culturally sensitive, and future research should be carried out on the cultural adaptation of EMDR therapy. In this regard, a recent study by Khan et al. [19] adapted EMDR therapy for post-traumatic stress disorder in Pakistan. Methodological adaptations of EMDR in similar contexts have also been limited, focusing primarily on modifications to the history taking, preparation, and assessment phases [31, 37–40].

The scarcity of research on adapting and clinically testing EMDR therapy in Pakistan, particularly in relation to online delivery formats and recently introduced the DeprEnd EMDR therapy protocol, is largely attributable to the relatively recent introduction of EMDR therapy in the country and the limited availability of trained clinicians. This gap is not merely methodological but reflects a broader neglect of implementation considerations that are central to mental health service delivery in Pakistan. In Pakistan, where mental health services are concentrated in urban centers and specialist availability is limited, reliance on exclusively in-person psychotherapy substantially restricts treatment reach [41]. Consequently, the limited exploration of online EMDR therapy is particularly consequential. However, online psychotherapy offers a viable pathway to reduce geographic, logistical, and financial barriers, and to extend specialist care to underserved populations. Evaluating online delivery is thus not a peripheral technical issue but a core implementation question, especially for interventions intended to be scalable and sustainable in real-world clinical systems. Within this context, the DeprEnd EMDR therapy protocol represents a particularly important case, as it remains insufficiently studied in terms of cultural adaptation and clinical validation in Pakistan. The cultural and methodological adaptation of the DeprEnd protocol has been reported in detail in a separate peer-reviewed publication [42]. Accordingly, the present manuscript focuses on the subsequent pilot feasibility assessment and randomized controlled evaluation of the adapted intervention, with a concise summary of the adaptation process provided solely to maintain conceptual continuity.

#### Theoretical basis of study

**Fig. 1:**
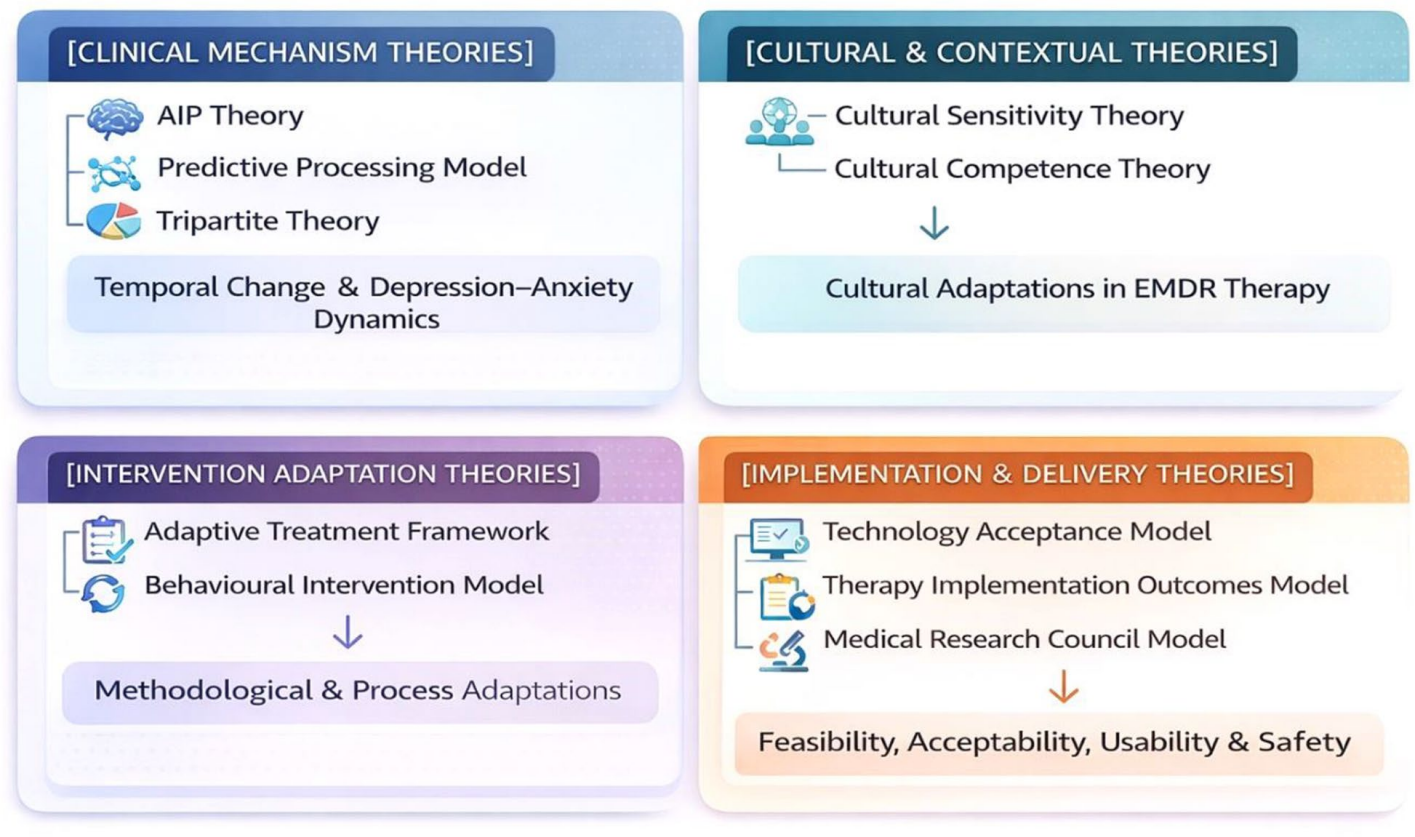
A conceptual grid of theories informing this study

Figure 1 presents relevant theories in a grid format, mapping their relevance to the constructs of this study. In the top-left quadrant, the first three theories address the clinical mechanism of EMDR therapy and depression-anxiety reciprocal links. To the right are theories on the cultural adaptation of EMDR therapy. The bottom row contains theories on methodological adaptation (left) and theories on feasibility, acceptance, and clinical effectiveness (right).

### Objectives {7}

Against this background and the aforementioned research gaps, the present study has the following three objectives:

1. To develop an adapted DeprEnd EMDR therapy protocol that is culturally and methodologically appropriate for the treatment of major depressive disorder in Pakistan.
2. To assess feasibility and acceptance of the adapted EMDR therapy for its delivery through in-person and online modes in Pakistan.
3. To assess the clinical effectiveness of the adapted DeprEnd EMDR therapy protocol across in-person and online settings.

Table 1 further provides the alignment of research objectives with the theoretical background of the study. The first objective of this study is grounded in theories such as Cultural Sensitivity Theory and the Adaptive Treatment Framework. These theories provide the conceptual basis for two premises: (a) therapies should be adapted both culturally and methodologically to meet the needs of clients and therapists, and (b) guidelines are required to identify the specific cultural and methodological elements to be included in adapted EMDR therapy. The second objective is guided by theories of Technology Acceptance and Therapy Implementation Outcomes. These models emphasize that user acceptance is a critical factor in determining the successful implementation of therapy. Moreover, understanding how users perceive and accept a therapy determines its feasibility. Finally, the third objective is theoretically anchored in the Adaptive Information Processing model, which explains symptom reduction through memory reconsolidation, while the Tripartite Model provides a complementary framework for examining depressive-anxiety symptomatology.

**Table 1:** Aligning study objectives and theoretical background

Research objectives	Theoretical basis
To develop an adapted DeprEnd EMDR Therapy protocol that is culturally and methodologically appropriate for treatment of Major depressive disorder in Pakistan	i. Cultural Sensitivity Theory [43] ii. Cultural Competence Theory [44] iii. Adaptive Treatment Framework [45] iv. Behavioral Intervention Model [46]
To assess feasibility, and acceptance of the adapted EMDR therapy for its delivery through in-person and online modes in Pakistan	i. Technology Acceptance Model [47] ii. Therapy Implementation Outcomes Model [48] iii. Medical Research Council Model [49]
To assess the clinical effectiveness of the adapted DeprEnd EMDR therapy protocol across in-person and online settings	i. Adaptive Information Processing Theory [50] ii. Predictive Processing Model of EMDR [51] iii. Tripartite Theory [52]

Taken together, the theoretical models outlined above formed an integrated explanatory foundation for the objectives of this study. Cultural Sensitivity and Adaptive Treatment frameworks justify the need for modifying the DeprEnd EMDR protocol to fit the Pakistani sociocultural and clinical context. Technology Acceptance and implementation models explained the conditions under which the adapted intervention can be practically and acceptably delivered across in-person and online modalities. Finally, the Adaptive Information Processing and Tripartite models provide a mechanistic rationale for expected symptom change in depression and its comorbid anxiety. This theoretical integration ensures that each research objective and corresponding hypothesis is conceptually grounded rather than merely empirically motivated.

## Hypotheses of study

Since the second phase of this study deals with clinical effectiveness, the following directional hypotheses will be tested:

i. The adapted EMDR therapy will demonstrate a high level of feasibility, usability, acceptance, technical operability, and safety when delivered through in-person and online modes.
ii. The online delivery of the adapted EMDR therapy is non-inferior to the in-person mode for treating major depressive disorder in Pakistan
iii. The adapted EMDR therapy will bring about significant temporal reduction in the major depressive disorder symptoms across in-person and online modes.
iv. The major depressive disorder symptoms are reciprocally linked to the comorbid generalized anxiety disorder symptoms in a manner that treatment conditions moderate this relationship.

### Trial design {8}

This study employs an exploratory sequential mixed-methods design [53] comprising three separate phases. The phase one focuses on the cultural and methodological adaptation of the DeprEnd EMDR therapy protocol using qualitative exploratory methods, including expert consultations and thematic analysis. This phase has been previously published in a dedicated peer-reviewed article [54] and here it is summarized only to provide continuity for phases two and three. The phase two consists of a quantitative pilot feasibility study by using a single-group pre- and post-test design [55] to assess feasibility, acceptability, safety, and preliminary symptom change following delivery of the adapted intervention. Finally, phase three comprises a multi-center, two-arm, single-blind randomized controlled trial designed to compare in-person mode with online by using a non-inferiority framework. Randomization, blinding, and hypothesis testing apply exclusively to phase three, whereas phase two is intended for feasibility assessment and intervention refinement.

## Methods: participants, interventions, and outcomes

### Study setting {9}

The present study will be conducted across multiple settings corresponding to the sequential three phases of the research.

i. Phase one will be conducted through a narrative literature review, expert consultations (involving EMDR therapists) based in Pakistan. These activities will be carried out through a combination of in-person meetings and secure online platforms, depending on participant availability.
ii. Phase two will be implemented in both online and in-person clinical settings at the selected clinics or psychotherapy centers located in the three selected cities in Pakistan, where the adapted DeprEnd EMDR therapy protocol will be delivered by four EMDR certified therapists. Data collection during this phase will occur through clinical assessments and standardized self-report measures.
iii. Finally, phase three will be conducted as a multi-center randomized controlled trial study implemented in both online and in-person clinical settings at the selected clinics or psychotherapy centers located in the three selected cities in Pakistan, where the adapted DeprEnd EMDR therapy protocol will be delivered by four EMDR certified therapists. Data collection during this phase will occur through clinical assessments and standardized self-report measures. During this phase, process fidelity and adherence to the adapted DeprEnd EMDR protocol will be monitored at mid of therapy.

#### Research framework of study

**Fig. 2:**
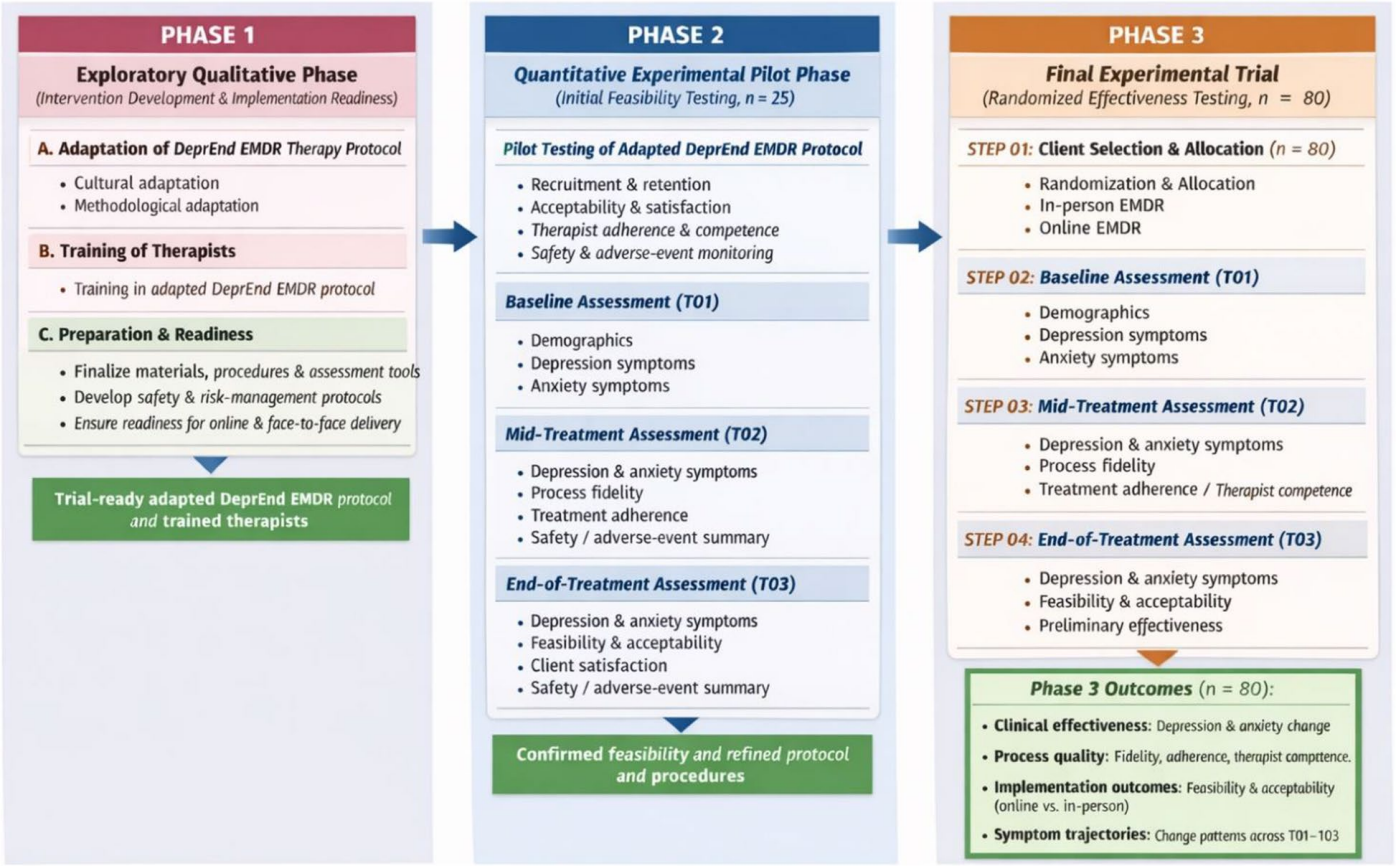
Research framework of study

Figure 2 illustrates a three-phase horizontal flow diagram. Phase one (Exploratory Qualitative Phase) presents the adaptation of the DeprEnd EMDR therapy protocol and preparation/readiness activities. This phase has been published in detail elsewhere [54].

Phase two (Quantitative Experimental Pilot Phase) shows the pilot testing process among a sample of *n* = 25. It includes baseline (T01), mid-treatment (T02), and end-of-treatment (T03) assessments, capturing dimensions of feasibility, acceptability, symptom measures, and client satisfaction. Phase three (Final Experimental Trial) includes a sample of *n* = 80 selection and allocation, repeated assessments (T01, T02, and T03), and ends with the outcomes box, summarizing the study’s outcomes such as clinical effectiveness, process quality, implementation outcomes, and symptom trajectories. Arrows between phases of the study highlight the sequential progression in the study from protocol development through pilot testing to full trial evaluation.

### Clients selection and sample size {14}

The study focuses on individuals with MDD in Pakistan, specifically selecting geographically accessible participants residing in the major cities of Rawalpindi, Islamabad, and Peshawar. Recent literature cites various studies such as by Ishtiaq et al. [56], Noor et al. [57] and Umar et al. [58] on the high prevalence of depressive disorders in these three cities of Pakistan. However, since the exact number of MDD cases in these three major cities is unknown, a more customized, multi-stage sampling procedure has been adopted.

Initially, three rehabilitation centers located in the selected cities will be randomly but purposively picked for recruiting patients. The centers will not be chosen fully at random; rather, the characteristics of the vicinity and patient flow into centers will be considered. Within each selected center, clients will be recruited using a sequential consecutive sampling technique with a rolling enrollment strategy. This approach involves selecting every eligible patient who meets the inclusion criteria until the required sample size is achieved [59]. The estimated sample size of (*n* = 80) was calculated by using the below given approximation method for two independent group means, as recommended in the existing literature by Sakpal [60] and Chow [61]:

$$n=\frac{\left({Z}_{\frac{\alpha }{2}}+{Z}_{\beta }{)}^{2}\cdot (2{\sigma }^{2}\right)}{\left({M}_{1}-{M}_{2}{)}^{2}\right.}$$

where.

*n*= is the estimated sample size.

Zα/2 = is the Z-score matching to the desired α level (for e.g., 1.96 for 95% confidence).

Z*β* = is the Z-score matching the desired power level (for e.g., 0.84 for 80% power).

σ^2^ = is the pooled variance of two groups.

µ​_1_ - µ​_2_= are the expected difference in means of depression scores.

A two-sided significance level of α = 0.05 was selected, consistent with conventional recommendations for clinical trials, resulting in α/2 = 0.025 and a corresponding Z-score of 1.96. Statistical power was set at 80% (1 − β = 0.80), yielding β = 0.20 and a corresponding Z-score of 0.84.

Based on previously published depression trials utilizing the Hamilton Depression Rating Scale-17 [62], a pooled standard deviation (σ = 8 points, variance = 64) for change scores was assumed. An expected mean difference of five points of the scale between the two treatment groups was specified, representing a clinically meaningful difference in depressive symptom severity. Substituting these values into the formula:

$$n=\frac{\left(1.96+0.84{)}^{2}\times (2\times {8}^{2}\right)}{{5}^{2}}$$

$$n=\frac{7.84\times 128}{25}$$

$$n=40$$

Hence, the estimated sample size required was 40 clients per treatment arm, resulting in a total sample size of 80 clients. Furthermore, to account for potential attrition, a dropout rate of 15% was assumed, consistent with longitudinal psychotherapy trials. The adjusted recruitment target was calculated using the formula:

$${n}_{\mathrm{initial}}=\frac{{n}_{\mathrm{target}}}{1-r}$$

$${n}_{\mathrm{initial}}=\frac{80}{0.85}=94$$

Accordingly, approximately 94 clients will be recruited to ensure that at least 80 participants complete the trial and are available for primary analysis.

### Eligibility criteria {10}

Each consecutive client in the selected centers will undergo Structured Diagnostic Interview for Screening DSM-5 (TR) Disorders [63] for an initial MDD diagnosis. Additionally, patients will be assessed for eligibility based on the below given inclusion criteria:

i. The clients should exhibit symptoms of MDD.
ii. Male and female clients will be selected equally.
iii. Clients between the ages of 20 and 50 will be selected. It was because this study is not on children or older clients.
iv. Clients should preferably be "treatment-naive," meaning they have no recent history of any psychotherapy or medication. This ensures that the adapted EMDR therapy can be implemented effectively without interference from previous therapies and to avoid confounding effects.
v. There must be no significant neuropsychological or cognitive disorders among patients.
vi. MDD with comorbid Anxiety is permitted in this study; however, clients should not have any other comorbid symptoms.

### Recruitment {15}

Clients will be recruited through professional therapeutic networks across participating cities. The principal investigator has conducted preliminary site visits to psychotherapy clinics in selected Pakistani capital cities to establish collaborations. Patient recruitment will follow a consecutive rolling enrollment strategy at each participating clinic. All prospective patients will undergo:

1. An initial diagnostic interview for MDD symptomatology verification.
2. Comprehensive eligibility assessment using study criteria.

Recruitment will continue sequentially until the target sample size (*n* = 80) is achieved, with an anticipated enrollment rate of 8–10 clients per month. Immediately following selection, participants will begin their allocated treatment (online or in-person EMDR). This consecutive enrollment and immediate treatment initiation process will be maintained consistently across all study sites until complete sample acquisition.

### Who will take informed consent? {26a}

Following initial screening interviews and administration of the Hamilton Depression Rating Scale-17 (score ≥ 20) and Generalized Anxiety Disorder Questionnaire-IV (score ≥ 5.7), eligible clients will undergo final eligibility assessment (see Eligibility Criteria). Therapists will notify all selected clients of their inclusion in the study. Clients will then receive the study information and consent materials. The therapists will obtain written informed consent from willing clients.

### Additional consent provisions for collection and use of participant data and biological specimens {26b}

Since the study protocol does not include the collection of any extra participant data or biological samples, additional consent procedures are not required.

## Assignment of interventions: allocation

### Sequence generation {16a}

**Fig. 3:**
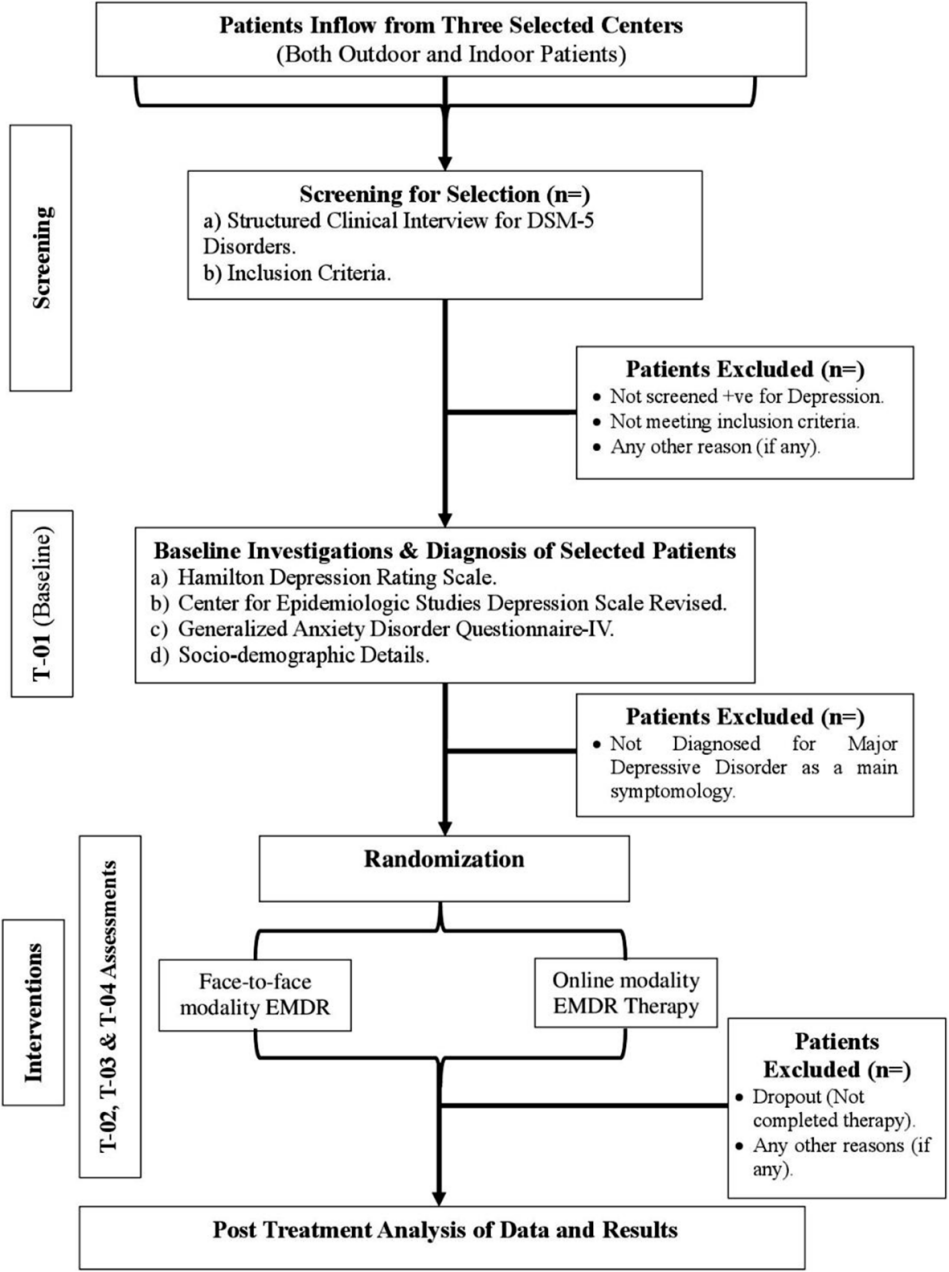
CONSORT flow chart of randomization

A covariate-adaptive randomization (minimization) approach with a random element will be employed to allocate participants in a 1:1 ratio to the intervention arms. This method is specifically designed to maintain balance across key prognostic variables in trials with moderate sample sizes and multiple baseline covariates, while preserving allocation unpredictability [64, 65]. Randomization will be implemented centrally using a pre-specified Python-based algorithm, executed by an independent research assistant following completion of baseline assessments. The algorithm evaluates imbalance across predefined covariates—including study site, gender, baseline depression severity, and psychiatric comorbidity—and assigns participants to the treatment arm that minimizes overall imbalance, with a probabilistic element (i.e., less than 100% deterministic assignment) to reduce predictability. This approach avoids the limitations of excessive stratification and fixed block randomization while ensuring transparent, reproducible, and auditable allocation. A detailed description of the randomization algorithm, operational procedures, and audit trail is provided in Supplementary File A, and Fig. 3 presents the CONSORT flow diagram illustrating participant allocation.

### Concealment mechanism {16b}

An independent research assistant will generate the randomization sequence using covariate-adaptive algorithms accounting for age, gender, depression severity, and comorbidity, with a 1:1 allocation to treatment groups. The randomization sequence will be securely stored in password-protected documents accessible exclusively to the principal investigator and the designated research assistant, with access logs maintained for auditing purposes. Allocation concealment will be ensured until assignment, and outcome assessors and data analysts will remain blind to group allocation throughout the trial. Due to the nature of the intervention, blinding of clients to treatment delivery mode (online vs. in-person EMDR) is not feasible. Group assignment will be implemented electronically via either OpenClinica (https://www.openclinica.com) or Sealed Envelope (https://www.sealedenvelope.com) platforms, which reveal only the immediate next assignment following completion of baseline assessments. This dual protection system—combining restricted access to master randomization lists with electronic allocation masking—ensures compliance with CONSORT and SPIRIT standards for allocation concealment.

### Implementation {16c}

Following eligibility confirmation and baseline assessments (T_1_), randomization will be executed through the designated electronic platform (OpenClinica/Sealed Envelope) by a research assistant who will not be involved in outcome measurement. The online system will be connected to the therapists’ and patients’ emails. It will automatically notify the treating therapist of the allocated intervention mode (online or in-person) and inform the patient via secure messaging, while maintaining the blinding of outcome assessors. To ensure protocol fidelity, therapists will receive arm-specific procedure checklists corresponding to their notification. The principal investigator will conduct weekly cross-checks between the electronic allocation records and physical randomization documents to detect and rectify any discrepancies.

## Assignment of interventions: blinding

### Who will be blinded {17a}

Due to the single blind-nature of intervention, complete blinding is not feasible for clients or therapists regarding delivery mode (online vs. in-person EMDR). However, therapists will remain blind until after completing the initial consultation with clients. Outcome assessors (research assistants) and data analysts will maintain full blinding throughout the trial. To minimize bias:

1. Outcome assessors will perform clinical evaluations before therapy sessions begin and without access to subsequent treatment setting information.
2. Data analysts will work with de-identified datasets using group codes.
3. For fidelity analysis, independent reviewers will assess edited audio/video recordings with all platform-specific references removed.
4. Blinding success will be empirically evaluated at the study midpoint by asking blinded personnel to guess group allocations, with ≤ 60% correct guesses indicating adequate maintenance of blinding.

### Procedure for unblinding if needed {17b}

Unblinding will be permitted only in exceptional circumstances (e.g., severe destabilization, emotional dysregulation, or psychiatric deterioration) or if critical data errors emerge during post-assessment analysis. The process will be initiated either by the treating therapist (for clinical emergencies), or the research assistant handling data (for analytical issues) via a secure email request to the principal investigator. Upon receipt, the principal investigator will:

1. Verify the necessity through documented clinical justification or data error reports.
2. Disclose only the minimally required treatment allocation or data details to the requesting party.
3. Record the unblinding event with timestamp, rationale, and disclosed information in the trial’s master log.

Notably, patients will not be informed of their group allocation unless medically imperative (e.g., for subsequent emergency care). All unblinding events will be reported to the ethics committee as protocol deviations.

## Interventions

### Explanation for the choice of comparators {6b}

The comparator is the in-person delivered EMDR therapy and is selected as the active control because it represents the current evidence-based standard of care for the treatment of depressive disorder [66]. Online EMDR therapy is an experimental intervention under investigation for non-inferiority. This design allows determining whether the novel online delivery method preserves therapeutic efficacy while offering potential accessibility benefits.

### Intervention description {11a}

This trial employs the evidence-based DeprEnd EMDR therapy protocol for depressive disorders [67]. This protocol is specifically optimized for depressive disorders through modifications in:

1. History-taking (focused on depressive triggers vs. trauma),
2. Preparation (enhanced psychoeducation on depression maintenance), and
3. Installation (targeting depressive cognition reprocessing) [21].

The DeprEnd EMDR therapy protocol has been culturally and methodologically adapted for the Pakistani context, and the full adaptation process has been published elsewhere [42]. In the present trial, both study arms follow the same adapted protocol, differing only in mode of delivery. The intervention will be delivered over 10–14 weekly sessions, depending on individual clinical presentation and treatment response. Each session will last approximately 60–90 min, consistent with standard EMDR therapy practice.

1. In-person EMDR therapy will be delivered by EMDR-certified therapists in selected psychotherapy clinics. Sessions will follow the adapted DeprEnd EMDR protocol using standard face-to-face procedures, including therapist-guided bilateral stimulation and adherence to protocol fidelity requirements.
2. Online EMDR therapy will be delivered via the BilateralBase platform, a validated telehealth system developed in the UK [68]. Therapists and clients will connect synchronously via secure video conferencing using a computer or mobile device. The platform provides visual and auditory bilateral stimulation, real-time therapist guidance, and session structuring consistent with in-person EMDR delivery. Session recordings are enabled for fidelity monitoring, with appropriate consent and ethical safeguards. Apart from delivery modality, the content, session structure, and therapeutic procedures remain equivalent to those used in the in-person arm.

### Criteria for discontinuing or modifying allocated interventions {11b}

Since participation in this study is voluntary, patients may discontinue the intervention at any time, as explained during the informed consent process. From a clinical perspective, the study intervention will be discontinued under the following circumstances:

1. Emergence of severe adverse reactions such as sustained dissociation, suicidal ideation, or emotional dysregulation that temporarily make the patient unresponsive to EMDR therapy—in such cases, therapy will pause to focus on resource-building and stabilization before potentially resuming.
2. Severe non-compliance, defined as missing more than four consecutive sessions without contact.
3. Therapist deviation from the given therapy protocol, as identified during mid-therapy fidelity inspections.

At the time of discontinuation, the following data will be collected to assist in planning the selection of future patients:

1. The point in time when discontinuation occurred.
2. The reason(s) for discontinuation, and when possible, the primary endpoint assessment and existing depressive symptom severity.

### Strategies to improve adherence to interventions {11c}

To optimize adherence to the intervention protocols, several strategies will be implemented.

1. First, before beginning therapy sessions, all participating therapists will receive comprehensive training in the adapted DeprEnd EMDR protocol. This training will be given at least for a week, and it will include orientation about the adapted therapy protocol and practical demonstration of eight steps of the protocol. It will enable the therapists to learn how to administer the adapted therapy protocol.
2. Second, patients will undergo thorough psychoeducation both before and during treatment to enhance their motivation and therapy compliance. The psychoeducation will include guidance about EMDR therapy, trauma, depression, the significance of therapy, and various resources related exercises.
3. Third, midway through the study (after completing therapies for 40 patients), a comprehensive fidelity analysis will be conducted to assess overall adherence to the treatment process, with corrective measures implemented if any deviations are identified; throughout the therapy period, regular fidelity checks will also be performed through random video audits of sessions.
4. Fourth, a flexible scheduling system will accommodate reasonable timing adjustments, particularly for online therapies, which will be scheduled according to client preferences. For clients showing early signs of non-adherence (such as two consecutive missed sessions), follow-up mobile calls or in-person contacts will be made with the clients or their family members to address potential barriers and encourage continued participation. Additionally, clients will have the option to reschedule missed sessions within a reasonable timeframe to ensure continuity of care. This proactive approach aims to support clients in overcoming obstacles and maintaining engagement in their therapy.

### Relevant concomitant care permitted or prohibited during the trial {11d}

As specified in the eligibility criteria, patients should preferably be treatment-naive (with no recent psychotherapy or medication use) to minimize confounding effects. However, exceptions will be made for cases where patients experience severe dissociation, derealization, or emotional dysregulation that compromises therapy adherence. In such instances, concomitant antidepressant medications may be prescribed by a psychiatrist and will be carefully documented (including dosage and frequency). For patients already taking antidepressants at baseline, these medications will not be discontinued to avoid destabilization. The primary objective in these cases will be to establish emotional stability to enable continued participation in the study protocol. Throughout the trial, patients will receive psychoeducation emphasizing the importance of not initiating any new psychological interventions. These measures are designed to balance scientific rigor with ethical clinical care while minimizing confounding variables.

### Provisions for post-trial care {30}

All trial participants will have access to appropriate post-trial care based on their clinical needs. For responders to therapy (defined as ≥ 60% reduction in HAM-D-17 scores), follow-up sessions will be offered to consolidate treatment gains, consisting of two monthly booster EMDR sessions (in-person or online, per their assigned study arm). Non-responding patients will be referred to stepped-care options, including medication management or alternative evidence-based psychotherapies, such as Cognitive Behavioral Therapy, through the network of collaborating psychiatric clinics.

### Outcomes {12}

#### Primary outcome

The primary outcome of the study is to study the change in depressive symptom severity. It will be assessed by using the Hamilton Depression Rating Scale-17 [69]. Depression assessments will be conducted at baseline (T01), mid-treatment (T02), end-of-treatment (T03), and follow-up (T04). The primary endpoint for evaluating clinical effectiveness and non-inferiority is the change in depression scores from baseline (T01) to end-of-treatment (T03).

#### Secondary outcomes

i. Anxiety symptom severity will be assessed by using the Generalized Anxiety Disorder Questionnaire-07 [70]. It will be assessed at baseline (T01), mid-treatment (T02), end-of-treatment (T03), and follow-up (T04).
ii. Feasibility, usability, acceptance, and safety of EMDR therapy will be evaluated by client retention rate [71], therapists adherence scale [72], Mobile Mental Health App Usability Questionnaire [73], User Acceptance of Technology Questionnaire [74] and Perceived Online EMDR Safety Scale [75], respectively.

### Participant timeline {13}

**Fig. 4:**
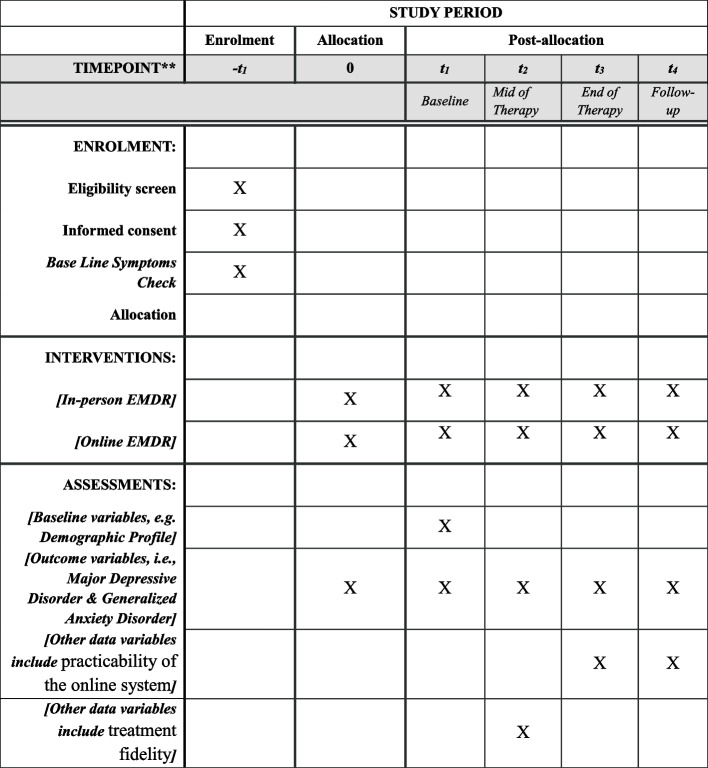
Patients’ timeline and schedule for enrollment, assessments, and intervention

The participant timeline is presented in Fig. 4 on the next page. Patient enrollment will occur at the T_1_ timepoint, which will also include the informed consent process, initial diagnostic interviews, and baseline symptom assessments. Following enrollment, patients will be allocated to either online EMDR or in-person EMDR therapy, with this group assignment maintained throughout the study duration. Regarding assessments:

1. Demographic characteristics and baseline clinical features will be collected at therapy initiation (T_1_).
2. Primary outcome measures (depression and comorbid anxiety symptoms) will be assessed at four key timepoints: baseline (T_1_), mid-therapy (T_2_), therapy completion (T_3_), and follow-up (T_4_) to evaluate temporal patterns of symptom reduction.
3. Treatment fidelity evaluations will be conducted at the mid-therapy point (T_2_) through standardized protocol adherence checks.

## Data collection and management

### Plans for assessment and collection of outcomes {18a}

Primary outcomes (depression severity assessed) and secondary outcomes (anxiety symptoms measured) will be collected at four timepoints: baseline (T1), mid-therapy (T2), post-intervention (T3), and 2-month follow-up (T4). All clinical ratings will be administered by trained research assistants who are blinded to treatment allocation. The feasibility and acceptability of the online platform will be evaluated using three validated instruments, as outlined in the protocol. Treatment fidelity and therapist competence will be assessed through both qualitative and quantitative methods, also detailed in the protocol. Data integrity will be ensured through automated checks for missing values and outliers, complemented by audits conducted by the statistical analyst. Any discrepancies will prompt source document verification by an independent monitor or research assistant.

### Plans to promote participant retention and complete follow-up {18b}

To maximize participant retention and follow-up completion, a multipronged strategy will be employed, including:

1. Regular check-in calls to address logistical or technical barriers.
2. Flexible scheduling options, including evening and weekend sessions to accommodate working participants.
3. A three-tiered follow-up procedure for missed appointments:
  Day 1: Email reminder.
  Day 3: Follow-up mobile/telephone call by a research assistant.
4. Dedicated “therapy booster” sessions for patients showing signs of disengagement (defined as ≥ 2 missed appointments).
5. Monthly retention reports, reviewed by the principal investigator to identify trends and adjust engagement strategies as necessary.

### Data management {19}

All study data will be managed using a tiered framework. Each recruited participant will be assigned a unique identification number. Physical data collected on paper will be securely stored in the principal investigator’s university archive, which is restricted and access-controlled. All study data will be archived for three years following the completion or premature termination of the trial. De-identified datasets will be retained for three years post-trial to allow for potential secondary analyses or regulatory audits. Only designated research assistants will be authorized to record and analyze data, and no other individual will be permitted to access or handle it. Role-based access permissions will be implemented, with the principal investigator having full access and research assistants granted access only to specific forms relevant to their responsibilities. All databases, including online, e.g., Google Drive, or in-system, e.g., Microsoft Excel and SPSS files, will be secured using two-factor authentication. Furthermore, Data use agreements will be mandatory for all third parties requesting access to the data, ensuring strict adherence to confidentiality and ethical standards.

### Confidentiality {27}

All data will be handled with the utmost confidentiality and will be accessible only to authorized personnel, such as the principal investigator. All participant data will be pseudo-anonymized, meaning that individuals will be identified only by a unique participant number. Any data used for publication or presentation will be fully anonymized, and only aggregated data will be reported to protect individual identities. The digital systems used for data management will include automatic session timeouts after ten minutes of inactivity and a login attempt limit of three tries to enhance data security and prevent unauthorized access.

### Plans for collection, laboratory evaluation and storage of biological specimens for genetic or molecular analysis in this trial/future use {33}

Not applicable.

### Ancillary and post-trial care {34}

All trial participants will receive appropriate post-trial care tailored to their clinical outcomes. Responders to therapy, defined as those achieving a ≥ 60% reduction in HAM-D-17 scores, will be offered two monthly booster EMDR sessions (delivered in-person or online, consistent with their assigned study arm) to reinforce treatment gains and reduce relapse risk. Non-responders will be referred to stepped-care alternatives, including medication management or evidence-based psychotherapies such as cognitive behavioral therapy, through the study’s network of collaborating psychiatric clinics. Referrals will include a summary of the participant’s trial data, shared with consent, to ensure continuity of care. For ancillary care, any unrelated medical or psychological needs identified during the trial will be addressed via referrals to local healthcare providers, with costs covered by [specify funding source: study sponsor, institutional support, or participant insurance]. Participants will be informed of these post-trial and ancillary care provisions during the consent process, including any limitations or exceptions.

## Methodology on treatment fidelity

Treatment fidelity will be established both qualitatively and quantitatively by adhering to the recommendations outlined by Dorsey et al. [76]:

1. The psychotherapy sessions will be video recorded and reviewed by two EMDR therapy experts. Moreover, quantitative ratings of recorded sessions will be done by using predefined rating scales such as the EMDR Fidelity Rating Scale [77].
2. The therapist’s competence will be assessed by self-reported rating scales such as the Instrument for Assessing Therapist Competence in Global Mental Health [78].
3. Finally, patients’ satisfaction and perception about adapted DeprEnd EMDR therapy protocol will be assessed by Scale for Patient Experience of Online EMDR therapy [79] and Satisfaction with Therapy Scale [80].

## Statistical methods

### Statistical methods for primary and secondary outcomes {20a}

All quantitative analyses in Phase two and Phase three will be conducted using appropriate statistical methods aligned with the study design, outcome structure, and hypotheses. Analyses will be performed using standard statistical software (e.g., SPSS and Python 3.10+, standard statistical libraries). Phase two analyses will focus on feasibility, acceptability, safety, and preliminary symptoms change following delivery of the culturally adapted DeprEnd EMDR protocol. Feasibility indicators will be summarized using descriptive statistics, and a one-sample t-test with 95% confidence intervals and significance levels. Repeated-measures General Linear Models will be used to determine temporal symptoms changes. These analyses will be exploratory and will not be intended to establish definitive clinical effectiveness.

Phase three analyses will be conducted within a randomized controlled trial framework. First, non-inferiority is evaluated by comparing the estimated 95% confidence interval for the between-group difference to a pre-specified margin; if the upper bound lies entirely below the margin, non-inferiority is concluded [81]. For the primary outcome, non-inferiority is based on the between-group difference in change scores on the Hamilton Depression Rating Scale-17 from baseline (T1) to the end of treatment (T3), calculated as online EMDR minus in-person EMDR. A non-inferiority margin (δ) of 3 points was pre-specified a priori, consistent with previously published non-inferiority trials that defined margins on this scale [82]. For secondary outcomes of anxiety, as assessed by the Generalized Anxiety Disorder-7, between-group differences in change scores will be examined using analogous comparative principles and reported with effect estimates and confidence intervals. A non-inferiority margin (δ) of approximately 2.5 points on this scale was pre-specified a priori, informed by clinically meaningful score differences used in prior non-inferiority psychotherapy trials [83].

Furthermore, following the procedures outlined by George and Mallery [84], General Linear Model Repeated Measures (GLM-RM) analyses will be conducted to examine longitudinal changes in major depressive disorder (MDD) and comorbid anxiety symptoms across assessment time points. The GLM-RM framework will be used to test the main effect of time, the main effect of treatment group (face-to-face vs. online EMDR), and the group × time interaction, which represents differential change over time between treatment modalities. Where significant effects are observed, planned post-hoc comparisons with Bonferroni correction [85] will be performed to examine changes in symptom severity between baseline (T01) and end of treatment (T04).

Finally, to account for repeated observations nested within individuals and to examine individual variability in symptom trajectories, hierarchical linear modeling (HLM) using maximum likelihood estimation will be employed, following the analytic guidelines described by Garson [86]. In these models, Level-1 will represent repeated symptom measurements over time, and Level-2 will represent individual participants. Time will be operationalized as the number of treatment sessions from baseline to follow-up and coded sequentially (e.g., 1, 2, 3, 4, 5…). Predictor variables will be entered as covariates and examined as fixed effects (representing average effects across participants) and, where appropriate, as random effects (representing individual variability in intercepts and slopes). Given the frequent co-occurrence of depressive and anxiety symptoms [87], reciprocal associations between depression and anxiety over time will be examined using HLM-based reciprocal association methodology [77, 80] and a 1–1–1 multilevel mediation modeling approach [88, 89].

### Interim analyses {21b}

The study will incorporate two strategically timed interim analyses to monitor trial progress and participant welfare. The first analysis, focused on safety monitoring, will be conducted after the initial cohort of 40 participants completes their mid-treatment assessments. This evaluation will systematically compare adverse event profiles between the online and in-person EMDR groups, with particular attention to clinically significant indicators such as dissociation rates. Should the analysis reveal substantially higher adverse events in either treatment arm—for instance, elevated dissociation occurrences in the online EMDR group—the protocol will be adaptively modified to incorporate additional stabilization sessions for affected participants while maintaining trial continuity.

The second interim analysis, designed to assess efficacy and futility thresholds, will occur when 60 participants (75% of the target sample) have completed their end-of-treatment evaluations. This analysis will employ O’Brien–Fleming statistical boundaries to determine whether the accumulating data demonstrates either compelling evidence of treatment success or insufficient likelihood of achieving meaningful results. For example, if the analysis reveals that online EMDR demonstrates near-equivalent efficacy to in-person delivery—as might be evidenced by merely a 0.5-point difference on the HAM-D-17 scale (*p* = 0.005)—the trial could be responsibly terminated early based on established efficacy. Conversely, should the data suggest minimal probability of ultimately proving non-inferiority, the futility provisions would be activated to conserve resources. These pre-specified analytical checkpoints are integrated into the study design to balance scientific rigor with ethical responsibility and operational efficiency.

### Additional analyses {20b}

Pre-specified subgroup analyses will examine treatment effect heterogeneity across key demographic and clinical variables, including age subgroups (20–35 vs. 36–50 years), baseline depression severity (HAM-D-17 scores 20–25 vs. ≥ 26), and trauma history (present vs. absent). These analyses will be conducted using interaction terms within mixed-effects models, with Bonferroni correction applied for multiple comparisons (adjusted *α* = 0.0167). Exploratory analyses will assess dose–response relationships using marginal structural models incorporating session attendance and homework completion rates. Sensitivity analyses will evaluate the robustness of findings by comparing per-protocol and intention-to-treat populations and by examining missing data patterns using Little’s MCAR test. For the online intervention arm, platform engagement metrics (e.g., login frequency and session duration) will be examined in relation to outcomes using partial least squares regression. All subgroup analyses will be interpreted as hypothesis-generating due to limited power, with effect sizes reported alongside 95% confidence intervals rather than relying solely on *p*-values.

### Methods for addressing non-adherence and missing data {20c}

To account for protocol deviations, primary analyses will employ both intention-to-treat and per-protocol approaches, with the intention-to-treat population including all randomized participants regardless of adherence and the per-protocol population restricted to those completing ≥ 80% of sessions with verified fidelity. For missing data, a tiered strategy will be implemented:

1. Continuous monitoring with initiated outreach after any missed assessment to minimize attrition.
2. Multiple imputation by chained equations using 50 imputed datasets incorporating baseline characteristics, treatment arm, and observed outcome trajectories as predictors.
3. Sensitivity analyses comparing results with pattern-mixture models that assume different missingness mechanisms (missing at random vs. not at random).

For participants who discontinue therapy but complete all outcome assessments, their available data will be included in the mixed-effects models used for longitudinal analyses.

### Plans to give access to the full protocol, participant-level data and statistical code {31c}

The full study protocol and its findings, in the form of a de-identified aggregate participant-level dataset, will be made publicly available six months after trial completion. However, these data will only be accessible as supplementary files accompanying published research articles and book chapters that present the findings of the study.

## Oversight and monitoring

The Trial Steering Committee will comprise both trial investigators and independent members, in accordance with Medical Research Council (MRC) guidance for independent trial oversight. The committee will be chaired by an independent senior academic with expertise in clinical trials and psychotherapy research, who is not otherwise involved in the conduct of the study. In addition to the independent chair, the Trial Steering Committee will include at least two further independent members, such as an external EMDR expert and an independent biostatistician, neither of whom will be involved in participant recruitment, intervention delivery, or data analysis. These independent members will provide objective oversight of trial progress, protocol adherence, safety monitoring, and ethical conduct. Trial-involved members of the Trial Steering Committee will include the principal investigator and a co-investigator, who will provide scientific input and operational updates. Supporting members, including research assistants, psychotherapists, a data analyst, and a consulting psychiatrist, will contribute to implementation and safety reporting but will not participate in independent decision-making. The Trial Steering Committee will operate under a formal written charter defining roles, responsibilities, meeting frequency, and decision-making processes, which will be finalized prior to trial initiation. This governance structure ensures appropriate independence, accountability, and robust oversight throughout the study.

### Composition of the data monitoring committee, its role, and reporting structure {21a}

The Independent data monitoring committee will consist of the principal investigator, a statistician, an EMDR therapy expert unaffiliated with this trial, and a patient representative with lived experience of depression. The committee will function under a formal charter that clearly defines its mandate. Its responsibilities include:

1. Conducting quarterly blinded reviews of adverse event reports and unblinded interim efficacy analyses in accordance with O’Brien–Fleming boundaries.
2. Evaluating protocol deviations that may affect data integrity.
3. Issuing recommendations regarding trial continuation, modification, or termination based on safety and efficacy considerations.

### Adverse event reporting and harms {22}

All adverse events will be recorded, classified, and reported by treating therapists according to the Common Terminology Criteria for Adverse Events version 5.0, using both severity grades (Grade 1 to 5) and related categories (definite, probable, possible, unlikely). All adverse events will be promptly reported to the principal investigator. Anticipated EMDR-specific adverse events—such as temporary emotional dysregulation or vivid dreaming—will be recorded in the trial’s adverse event log. To mitigate potential risks, therapists will implement several safeguards, for example, conduct pre-session safety screenings using the Columbia-Suicide Severity Rating Scale; ensure emergency psychiatric contacts are available at all clinics; and deliver post-session stabilization procedures for patients exhibiting any signs of acute distress.

### Frequency and plans for auditing trial conduct {23}

To maintain high standards throughout the study, a comprehensive quality assurance system will be established, incorporating multiple layers of oversight. The monitoring approach will combine scheduled reviews with responsive checks to ensure complete and accurate trial conduct. The quality control process will begin with quarterly centralized audits conducted by the research team. These systematic reviews will examine essential study documents, including signed consent forms, case report forms, and adverse event reports. A randomly selected 10% of all primary outcome data will be verified against original source documents during these audits to confirm accuracy. Additional targeted audits will be conducted at participating clinics if any concerns arise, such as higher-than-expected protocol deviations or irregular patterns in recruitment. These on-site reviews will focus on therapy session documentation and the implementation of appropriate randomization procedures to ensure strict adherence to the trial protocol.

### Plans for communicating important protocol amendments to relevant parties (e.g., trial participants, ethical committees) {25}

Any changes made to the study protocol will be clearly communicated to all relevant parties in a timely and organized manner.

1. If the changes are major, for example, affecting study design, participant safety, or eligibility criteria, they will be submitted for approval to the Research Ethics Review Committee and Institutional Review Board of the parent university before being put into practice.
2. Once an amendment(s) is approved, it will be shared with all personnel involved who will be notified by email within 24 h. Such personnel will be required to confirm they have received and understood the changes by signing an acknowledgment form.
3. ClinicalTrials.gov will be updated within 07 days of the amendment being approved, to ensure public transparency.
4. Every amendment and its related communication will be thoroughly documented, including the date of approval, the method and timing of dissemination (e.g., email or verbal), and confirmation that such changes have been understood by all personnel involved.

## Discussion

MDD represents a significant public mental health concern in Pakistan, with epidemiological data indicating a clinically substantial prevalence in the country [3, 5, 79]. Yet access to mental health care remains critically limited, particularly in rural areas [84]. This treatment gap highlights the need for culturally adapted interventions in Pakistan. It implies cultural and methodological adaptation of EMDR therapy in Pakistan is a necessity, and not an option. The unique cultural norms of Pakistani clients may require adaptations in the EMDR therapeutic techniques in order to align it with the local clinical setting. Keeping this in view, the present study employed a structured, three-phase framework to systematically adapt, pilot, and evaluate the DeprEnd EMDR therapy protocol in Pakistan across online and in-person delivery modalities. This phased approach integrates qualitative exploration, quantitative feasibility testing, and randomized effectiveness evaluation, thereby strengthening both internal validity and implementation relevance of the study.

Phase one emphasizes cultural and methodological adaptation, therapist training, and implementation readiness by successfully addressing known challenges in transferring evidence-based psychotherapies across contexts and delivery formats. Such preparatory work is critical for maintaining treatment fidelity and safety, particularly in digitally delivered interventions [90]. Phase two employs a pilot design (*n* = 25) with assessments at baseline (T01), mid-treatment (T02), and end-of-treatment (T03), allowing systematic evaluation of feasibility, acceptability, safety, and process quality. This structure is consistent with recommendations that pilot studies emphasize implementation outcomes rather than hypothesis testing [91]. The inclusion of mid-treatment process indicators supports ongoing fidelity monitoring, which is known to improve intervention quality and interpretability of effects [92]. The comprehensive T03 assessment strengthens confidence in protocol refinement prior to large-scale testing. Lastly, phase three extends the framework into a randomized effectiveness trial (*n* = 80), comparing online and in-person EMDR delivery. It reflects a hybrid effectiveness implementation perspective by allowing concurrent evaluation of clinical outcomes and implementation processes [93].

This study will play a pivotal role in advancing the mental healthcare system in Pakistan by directly addressing critical gaps in the availability, accessibility, and cultural appropriateness of treatments for MDD. Beyond its empirical contributions, this research has practical implications for mental health professionals engaged in psychotherapy and clinical research across low- and middle-income countries. By combining methodological approaches from both medical science and psychotherapy, the study sets the stage for transdisciplinary collaboration and innovation in the mental health field in Pakistan. Ultimately, it contributes to the long-term goal of building a more accessible, effective, and culturally responsive mental health infrastructure in Pakistan and potentially across other parts of South Asia.

## Trial status

The adaptation of the DeprEnd EMDR therapy protocol (version 2.0) was finalized on January 15, 2025. The trial is currently recruiting participants. Recruitment began on April 1, 2025, and is expected to be completed by the end of February 2026.

## Supplementary Information

Supplementary Material 1. Supplementary File A: Operational Procedure for Randomization and Allocation

## Data Availability

Due to ethical, legal, and intellectual property considerations, the following data will not be made publicly available: • The *DeprEnd—EMDR Therapy Protocol* that will be employed in this study represents the intellectual property of its original authors and is protected under copyright law. Therefore, it will not be publicly distributed. • To safeguard participant confidentiality, individual-level clinical data will not be disclosed. However, de-identified, aggregate-level data may be made available to qualified researchers upon reasonable request. Such requests will be reviewed on a case-by-case basis by the corresponding author, in accordance with the data protection regulations and ethical policies of the parent organization. Researchers interested in accessing such data will be encouraged to contact the corresponding author for further information.
