# Supplementary material for: Adapting and testing of DeprEnd EMDR therapy for major depressive disorder: a study protocol of mixed method randomized controlled trial
Source: Trials. 2026 Feb 16;27:234. doi: 10.1186/s13063-026-09543-4 (PMC13019998; doi:10.1186/s13063-026-09543-4)
Supplement: Supplementary file 1 — Supplementary Material 1. Supplementary File A: Operational Procedure for Randomization and Allocation [file 13063_2026_9543_MOESM1_ESM.docx]

**Supplementary File: A**

**Operational Procedure for Randomization and Allocation**

**Purpose of this file**

This document describes how randomization will be practically implemented during trial conduct, step by step, from the point at which a participant completes baseline assessment to the point at which the intervention arm is assigned. The procedure is designed to ensure balance, concealment, transparency, and auditability under routine clinical-research conditions.

Who will perform randomization and when

Randomization will be performed by an independent research assistant (RA) who will not be involved in:

- 1. participant recruitment,
  2. intervention delivery,
  3. outcome assessment, or
  4. data analysis.

Randomization will occur only after:

1. written informed consent is obtained, and
2. all baseline eligibility assessments are completed and verified.
3. No allocation decisions will be made at screening or consent stages.

**Information that will be required before randomization**

Before randomization, the following baseline data will be collected and entered into a secure dataset:

1. Study site (city/center)
2. Gender
3. Baseline depression severity (HAM-D-17 score, categorized as 20–25 or ≥26)
4. Presence or absence of psychiatric comorbidity

These data will be entered using a standardized baseline entry form to avoid ambiguity or subjective categorization.

**How the randomization will be executed (step-by-step)**

**Step 1: Initiating the process**

Once baseline data entry is completed, the recruiting clinician will notify the independent RA that the participant is ready for allocation. The clinician will have no access to the randomization process.

**Step 2: Running the algorithm**

The RA will run a pre-specified Python-based randomization script stored on a password-protected computer. The script will be finalized and locked prior to trial initiation.

The script will:

1. read the current cumulative allocation file,
2. incorporate the new participant’s baseline covariates,
3. calculate imbalance scores assuming assignment to each treatment arm.

**Step 3: Allocation decision**

The algorithm will assign the participant to one of the two intervention arms using a covariate-adaptive minimization rule with a random element:

1. the arm that minimizes imbalance will be favored,
2. a probabilistic element (e.g., 70–80% probability) will be applied to prevent predictability.

This approach will ensure both balance and allocation unpredictability.

**Step 4: Recording allocation**

Immediately after assignment:

1. The script will generate an allocation record containing:
   - participant study ID,
   - date and time of allocation,
   - baseline covariate profile,
   - assigned treatment arm.
2. this record will be automatically appended to a read-only audit log file.

**4. How allocation concealment will be maintained**

1. No randomization list will exist in advance.
2. The RA will see only the current participant’s allocation and will not have access to future assignments.
3. Therapists, investigators, and outcome assessors will not have access to:
   - the randomization script,
   - imbalance calculations, or
   - allocation sequences.

Allocation information will be disclosed only after assignment and only to personnel responsible for scheduling the intervention.

**5. How allocation will be communicated**

Following randomization:

1. the RA will inform the trial coordinator of the assigned intervention arm using a secure communication channel.
2. the trial coordinator will arrange scheduling of online or face-to-face EMDR therapy accordingly.
3. Outcome assessors will remain blinded to treatment allocation.

**6. Data storage and auditability**

1. Randomization files and audit logs will be:
   - password protected,
   - stored on encrypted devices,
   - backed up at regular intervals.
2. Each allocation will be timestamped and traceable.
3. Logs will be reviewed periodically by the principal investigator to confirm protocol adherence.
4. No manual editing of allocation files will be permitted.

**7. Contingency procedures**

In the event of technical difficulties (e.g., computer malfunction):

- randomization will be temporarily paused,
- no manual or alternative allocation method will be used,
- the issue will be resolved before recruitment resumes.

Any interruption or deviation will be documented and reported to the ethics committee if required.

**8. Rationale for this procedure**

This operational approach will:

- avoid excessive stratification and fixed block randomization,
- reduce the risk of allocation predictability,
- remain feasible in routine clinical settings,
- align with CONSORT and SPIRIT recommendations for psychotherapy trials.
